# Burden of community-acquired pneumonia, predisposing factors and health-care related costs in patients with cancer

**DOI:** 10.1186/s12913-018-3861-8

**Published:** 2019-01-14

**Authors:** Niklas Schmedt, Olivia Denise Heuer, Dennis Häckl, Reiko Sato, Christian Theilacker

**Affiliations:** 1 InGef – Institute for Applied Health Research Berlin, Berlin, Germany; 2 WIG2 - Scientific Institute for Health Economics and Health System Research, Leipzig, Germany; 3 Pfizer Inc., Collegeville, PA USA; 4 Pfizer Deutschland GmbH, Berlin, Germany

**Keywords:** Community-acquired pneumonia, Cancer, Epidemiology, Mortality, Germany, Healthcare costs, Retrospective studies, Incidence, Economics, Claims data

## Abstract

**Background:**

Data on the burden of community-acquired pneumonia (CAP) and health-care related costs in patients with cancer is scarce. We aimed to estimate the CAP incidence rate, mortality, and healthcare-related costs of CAP patients with different cancer subtypes in Germany.

**Methods:**

We used German health claims data of a representative sample of 4 million subjects to conduct cohort studies in patients with a new diagnosis of lung, hematological, breast, gastro-intestinal tract and renal/urinary-tract cancer and a comparator cohort without cancer between 2011 and 2015. CAP cases were identified in both the hospital and ambulatory care setting. Crude and age- and sex-standardized incidence rates (sIR) of CAP and mortality after CAP were calculated. To compare the health care-related costs of cancer patients with and without a diagnosis of CAP, a propensity-score (PS) matched control group was created.

**Results:**

The study population comprised of 89,007 patients with cancer. In lung cancer patients, the sIR was increased 21-fold compared to the control cohort. For the other cancer subtypes, the sIR was increased 4.3-fold (hematological malignancies) to 1.7-fold (breast cancer) compared to the control cohort. The 30-day mortality in CAP cases was highest in lung cancer patients with 20.0% and ranged from 7.2 to 18.5% in CAP cases with other cancer subtypes. The highest costs were observed in CAP cases with hematological malignancies with 28,969 € (SD 37,142 €) and the lowest in patients with renal/urinary tract cancer with 17,432 € (SD 19,579 €). The absolute difference in the mean overall costs between CAP cases and controls without CAP ranged from 4,111€ to 9,826€, depending on the cancer type. CAP-related costs were predominantly triggered by substantially elevated hospital costs in CAP cases.

**Conclusions:**

The incidence rate of CAP and related mortality is high in patients with cancer with strong variations by cancer subtype. Furthermore, CAP in cancer patients is associated with substantial direct excess costs.

**Electronic supplementary material:**

The online version of this article (10.1186/s12913-018-3861-8) contains supplementary material, which is available to authorized users.

## Background

Community-acquired pneumonia (CAP) is a significant cause of morbidity, mortality, and healthcare costs in Europe [1]. For Germany, a total of 271,352 hospitalized CAP cases in adults aged ≥18 years were reported in 2016, equating to an annual incidence of 393 per 100,000 persons [2]. In general, the risk for CAP is higher in older age groups and in patients with underlying comorbidities and immunocompromised states such as adults with cancer [1]. Pelton et al. [3] showed that the risk of all-cause pneumonia was 2 to 19 times higher in patients with at-risk or high-risk conditions for all-cause pneumonia, respectively, and that the risk steadily increased with a higher number of co-morbid conditions. In cancer patients, the incidence rate of pneumonia was increased approximately two-fold for children and adolescents (< 18 years) and more than threefold in all other age groups compared to healthy subjects [3]. Accordingly, pneumococcal and influenza vaccination in cancer patients is recommended in several guidelines, e.g. by the Advisory Committee on Immunization Practices, Infectious Disease Society of America or the German Standing Committee on Vaccination (“Ständige Impfkommision”, STIKO) [4–7].

In this context, it has to be noted that data on the burden of disease and the economic burden of CAP in a large subgroup of cancer patients is scarce for Germany and also for other Western countries. While Pelton et al. [3] demonstrated the increased risk of CAP in cancer patients, limitations of this study included not distinguishing between CAP and hospital acquired pneumonia, lack of differentiation between cancer subtypes, and the absence of data on associated healthcare-related costs for CAP. Against this background, we aimed to extend findings of previous studies examining the epidemiologic and economic burden of CAP in cancer patients by cancer subtypes in the German population. The current study estimated the CAP incidence rate, mortality, healthcare-related costs and risk factors for developing CAP in cancer patients.

## Methods

### Data source

The study was based on the InGef (Institute for Applied Health Research Berlin, formerly Health Risk Institute (HRI)) research database which includes longitudinal claims data of approximately 6.7 million subjects from approximately 64 statutory health insurance providers (SHIs) from all geographic regions in Germany. A dataset with a reduced sample size of approximately 4.6 million subjects has been created to obtain a representative sample of the total SHI population in Germany with regard to age and sex and was used for this study [8]. This sample covers approximately 5% of the total German population.

In brief, the InGef research database includes demographic data; claims data for ambulatory services and procedures according to the German uniform assessment standard (EBM, ‘Einheitlicher Bewertungsmaßstab’) and including the information on diagnostic certainty (verified, status post, exclusion or suspected); hospital data including admission and discharge dates, the main and secondary discharge diagnoses and codes for procedures conducted in hospital according to the German Procedure Classification (OPS, ‘Operationen und Prozeduren Schlüssel‘); drug prescription and dispensing data with the date of prescription and drug dispensation; reimbursed remedies and aids; and costs of each healthcare sector from the perspective of the German SHIs [8]. All diagnoses in the database are coded according the German modification of the 10th revision of the International Classification of Diseases (ICD-10 GM). Data contributing to the InGef database are stored at a specialized data center owned by SHIs providing data warehouse services. In the data center (acting as a trust center), data with respect to individual insured members, health care providers (e.g. physicians, practices, hospitals, pharmacies), and the respective SHI are anonymized.

### Study design and setting

We conducted cohort studies in patients with five cancer subtypes: lung cancer or cancer of airways, hematological malignancies, breast cancer, cancer of the gastro-intestinal tract, and renal cancer or cancer of the urinary tract.

To estimate the incidence rate of CAP in patients with specific cancer subtypes and to conduct nested case-control studies to identify factors associated with CAP, we created five cohorts of patients with an incident cancer diagnosis (cancer cohort) between 2011 and 2015. Subjects were eligible to enter the cohort, if they had (i) at least one diagnosis for the respective cancer subtype (see Additional file 1) between 2011 and 2015, (ii) valid information on age and sex, (iii) age of at least 18 years, (iv) continuous insurance of at least four quarters without any cancer diagnosis (baseline period), (v) no diagnosis of any pneumonia (ICD-10 GM codes, J12-J18) in the quarter before cohort entry and (vi) female sex (only for the analysis of breast cancer). All patients entered the cohort at the beginning of the quarter in which the incident cancer diagnosis was made (cohort entry date), if all inclusion criteria were fulfilled. Cohort exit was defined as the end of insurance (including death), the end of the observation period (31st December 2015) or the occurrence of CAP, whichever occurred first.

We compared the incidence rate of CAP in the cancer cohort to a control cohort without cancer. Subjects were eligible for the control cohort, if they had (i) continuous insurance of at least four quarters without any cancer diagnosis (baseline period), (ii) valid information on age and sex, (iii) age of at least 18 years, (iv) no diagnosis of any pneumonia in the quarter before cohort entry and (v) female sex (only for the analysis of breast cancer). All subjects entered the cohort after continuous insurance of four quarters, if all inclusion criteria were fulfilled. Cohort exit was defined as the end of insurance (incl. death), the end of the observation period, any cancer diagnosis or the occurrence of CAP, whichever occurred first.

In order to calculate the 30-day and one-year mortality, to calculate the proportion of patients with hospital readmission and to assess the healthcare-related costs after CAP diagnosis, a sub-cohort with CAP patients (CAP cohort) was selected from the respective cancer cohorts. Patients were eligible for the CAP cohort, if they had (i) at least one diagnosis of CAP (see case definition below) during follow-up in the respective cancer cohort and (ii) continuous insurance for at least 365 days or until death after the incident CAP diagnosis (observation period). All CAP cases were followed up from the incident CAP diagnosis (index date) until death or one year after the index date, whichever occurred first.

### Identification of CAP cases

CAP cases were defined as patients with a primary hospital diagnosis of pneumonia (ICD-10 GM codes, J12-J18) or a secondary hospital diagnosis in combination with a hospital admission diagnosis of pneumonia. Patients with a secondary hospital diagnosis indicating hospital-acquired pneumonia (ICD-10 GM code U69.00) in a hospital stay with a duration of at least two days or a hospital discharge in 7 days prior to the index date were not considered as CAP case. In the outpatient setting, a prescription for an antibiotic (Anatomical Therapeutic Classification (ATC) codes J01AA*, J01CA* (excl. J01CA08), J01 CE*, J01CR*, J01DB*, J01 DC*, J01DD*, J01DE*, J01DH*, J01EE*, J01FA*, J01MA*, J05AB*) and at least one recorded claim for a chest x-ray, CT-scan or MRI in the same quarter were required.

### Risk factors for CAP

Risk factors for CAP were identified based on hospital and verified outpatient diagnoses as well as OPS codes subdivided into at-risk conditions and high-risk conditions (see Additional file 1)). At-risk conditions included chronic heart disease, chronic pulmonary disease (including asthma), diabetes mellitus and neurological disorders. High-risk conditions were defined as functional or anatomic asplenia, sickle cell diseases and other hemoglobinopathies, chronic severe liver disease, HIV infection, chronic renal failure or dialysis, autoimmune diseases, immunosuppressive treatment, solid organ or stem cell transplantation, congenital immunodeficiency, neutropenia/agranulocytosis. Cancer-specific risk factors included metastatic solid tumors, radiation therapy, cytotoxic chemotherapy, use of immunosuppressants and stem cell transplantation.

### Statistical analysis

#### Calculation of CAP incidence rate

The incidence rate of CAP per 100,000 person-years was calculated for each cancer cohort and the control cohort stratified by age group (18–59, 60–74 and 75+ years), and presence of additional risk factors for CAP (yes vs. no) within age-group, dividing the absolute number of cases by the amount of accumulated person time in the cohort of the respective stratum. For descriptive purposes, 95%-confidence intervals were provided for the incidence rates assuming a Poisson distribution [9]. To adjust for differences by age and sex, the incidence rate for CAP in each cohort was also standardized according to the age and sex distribution of the person time in the control cohort.

#### Calculation of mortality and hospital readmissions after CAP diagnosis

The 30-day and the one-year mortality after CAP in cancer patients was calculated stratified by age group (18–59, 60–74 and 75+ years) and presence of additional at-risk conditions for CAP (yes vs. no) as the percentage of patients who died within 30 days and 365 days after the first diagnosis of CAP. The date of death was defined as the date of disenrollment from the insurance with death as documented reason for disenrollment. Among hospitalized CAP cases, the percentage of patients with hospital readmission within 30 days after hospital discharge due to all causes and CAP was calculated.

#### Nested case-control analyses

To identify predisposing factors of CAP in cancer patients, we conducted nested-case control analyses in the respective cancer cohorts. For this purpose, CAP cases were selected from the respective cancer cohort. For each CAP case, a random sample of up to 4 controls was selected from the risk-set of the respective patient at the date of CAP diagnosis (index date) and matched according to calendar date (on a quarterly basis) and cohort entry date. The risk-set comprised all cancer patients in the respective cohort who were at risk to become CAP case at the index date, i.e. patients without CAP, without hospital discharge within 7 days prior to the index date and without hospitalization at the index date. Multivariable conditional logistic regression was used to estimate odds ratios (ORs) for risk factors of CAP prior to cohort entry and during follow-up. In a first step, bivariate ORs were calculated for each risk factor. All variables statistically significant associated with CAP were included in the multivariate model. A cut-off level of 5% was chosen to define statistically significant associations.

#### Comparison of costs and one-year survival in CAP cases and matched control groups

To compare the health care-related costs (overall and for the cost components of ambulatory care, hospital care, drugs, remedies and aids) of cancer patients with and without a diagnosis of CAP, a propensity-score (PS) matched control group was created. The source population for the controls consisted of all cancer patients in the cancer cohorts without CAP during follow-up. For each potential control, a random index quarter was assigned. Each potential control was eligible for analysis if he/she had continuous insurance for at least 365 days or until death after the random index date. The PS was calculated as the probability of being assigned to the CAP group depending on a set of given covariables including age, sex, 80 conditions associated with high costs from the perspective of the SHIs according to the German structure compensation scheme (Morbi-RSA), as well as the logarithm of the individual cost components of outpatient care, inpatient care, drugs, remedies and aids. For each CAP case in the respective cancer cohort, a control without CAP was matched by cancer subtype, time since incident cancer diagnosis and PS using nearest-neighbor matching, (1:1 matching). To guarantee a minimal observation period of at least one year, only CAP cases between 2011 and 2014 were considered.

After matching, the balance of the baseline costs (overall and for the cost components of outpatient care, inpatient care, drugs, remedies and aids) was checked. Sufficient balance of the baseline costs was assumed in case of standardized differences (SMD) ≤0.1 for the cost variables. For all outcome variables related to costs, the mean, median, minimum, maximum and the standard deviation was reported in CAP cases and the matched comparator group. To test for differences in means, the Mann-Whitney-U-test was used.

In addition, Kaplan-Meier curves were generated to display the one-year survival after diagnosis in CAP cases compared to a control group without CAP matched by age (± 1 year), sex, cancer subtype, and time since incident cancer diagnosis (1:1 matching). The log-rank test was used to test for significant survival differences. For this analysis, the index date was set to the exact date of diagnosis of the CAP case for both cases and controls.

All statistical analyses were conducted with SAS, version 9.4 (SAS Institute) and results are reported according to German Reporting Standard for Secondary Data Analyses [10].

## Results

### Study population

The source population of the study comprised approximately 4.6 million subjects from the InGef research database. Of those, 89,007 patients had an incident diagnosis of the respective cancer subtypes between 2011 and 2015. Diagnoses for cancer of the gastro-intestinal tract was the most common with 30.8%, followed by breast cancer (25.2%), hematological malignancies (17.2%), renal and urinary tract cancer (13.3%), and lung cancer (13.3%). There were more females (57.8%) and 36.4% of patients were in the age group 18–59 years, 35.9% in the age group 60–74 years, and 27.6% in the 75+ years age group. The mean age was 64.1 years (standard deviation (SD) 15.2 years). The characteristics of all cancer cohorts are displayed in Additional file 2.

### CAP incidence rate

The crude incidence rate of CAP varied substantially between the different cancer subtypes (Table 1). The highest was observed in lung cancer patients with 14,467 per 100,000 person years (95%-confidence interval [CI]: 13,832-15,125) and the lowest in breast cancer patients with 931 per 100,000 person years (95%-CI: 854–1013). The age and sex- standardized incidence rates (sIR) were substantially lower compared to the crude incidence rate for all cancer subtypes, but still varied substantially. In lung cancer patients, the sIR was increased 21-fold compared to the control cohort. For the other cancer subtypes, the sIR was increased 4.3-fold (hematological malignancies) to 1.7-fold (breast cancer). The incidence rate of CAP stratified by age and presence of underlying risk factors for CAP is available in Additional file 2.

**Table 1:** Crude and standardized incidence rates of CAP in the different cancer and comparator cohorts

	n cohort	n CAP cases	PY	IR per 100,000 PY	Lower 95% CI	Upper 95% CI
Lung cancer						
Crude IR	11,837	1945	13,444	14,467.4	13,831.5	15,125.0
Standardized IR^a^		1,256,235	13,188,819	9525.0	9508.4	9541.7
Hematological malignancies						
Crude IR	15,317	1002	33,662	2976.7	2795.2	3166.8
Standardized IR^a^		255,193	13,188,819	1934.9	1927.4	1942.4
Cancer of the gastro-intestinal tract						
Crude IR	27,423	926	58,752	1576.1	1476.2	1681.0
Standardized IR^a^		108,308	13,188,819	821.2	816.3	826.1
Renal cancer & cancer of the urinary tract						
Crude IR	13,197	532	29,431	1807.6	1657.3	1968.0
Standardized IR^a^		112,611	13,188,819	853.8	848.9	858.8
Control cohort (overall)						
Crude IR	3,192,611	59,581	13,188,819	451.8	448.1	455.4
Breast cancer						
Crude IR	22,450	534	57,359	931.0	853.7	1013.4
Standardized IR^a^		45,855	6,647,290	689.8	683.5	696.2
Control cohort (breast cancer)						
Crude IR	1,604,248	27,154	6,647,290	408.5	403.7	413.4

^a^According to the age and sex distribution of the person time in the comparator cohort, *CAP* Community-acquired pneumonia, *CI* Confidence interval, *IR* Incidence rate, *PY* Person year

### Mortality and hospital readmissions among CAP cases

Table 2 shows the 30-day and one-year mortality in CAP cases with different cancer subtypes between 2011 and 2014. The 30-day mortality in CAP cases was highest in lung cancer patients with 20.0% (95%-CI: 18.0–22.1%) and ranged from 16.9 to 18.5% in CAP cases with other cancer subtypes except for those with breast cancer. Here, the 30-day mortality was substantially lower with 7.2% (95%-CI: 4.8–10.4%). A similar pattern was observed for the one-year mortality which was highest in lung cancer patients (63.5%; 95%-CI: 61.0–65.9%) and lowest in breast cancer patients (19.8%; 95%-CI: 15.8–24.3%). The 30-day mortality and one-year mortality in CAP cases stratified by age group and presence of underlying risk factors for CAP is available in Additional file 2.

**Table 2:** 30-day and one-year mortality in CAP cases in different cancer subtypes

30-day mortality					
Cancer subtype	n CAP cases	n deaths	30-day mortality (%)	Lower 95% CI	Upper 95% CI
Lung cancer	1531	306	20.0	18.0	22.1
Hematological malignancy	732	124	16.9	14.3	19.9
Breast cancer	359	26	7.2	4.8	10.4
Cancer of the gastro-intestinal tract	666	123	18.5	15.6	21.6
Renal cancer & cancer of the urinary tract	371	67	18.1	14.3	22.4
One-year mortality					
Cancer subtype	n CAP cases	n deaths	365-day mortality (%)	Lower 95% CI	Upper 95% CI
Lung cancer	1531	972	63.5	61.0	65.9
Hematological malignancy	732	308	42.1	38.5	45.7
Breast cancer	359	71	19.8	15.8	24.3
Cancer of the gastro-intestinal tract	666	313	47.0	43.2	50.9
Renal cancer & cancer of the urinary tract	371	184	49.6	44.4	54.8

*CAP* Community-acquired pneumonia, *CI* Confidence interval

**Fig. 1:**
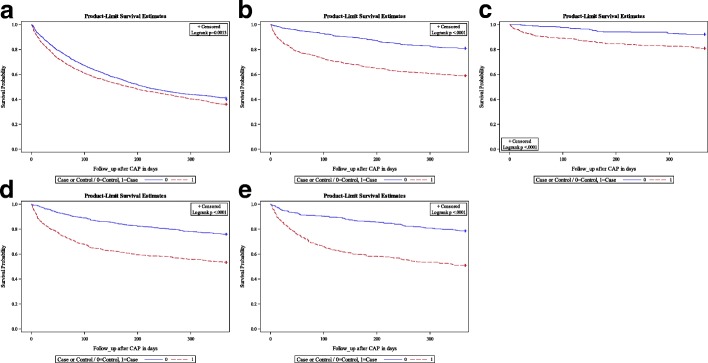
One-year survival in CAP cases and age and sex-matched controls with (**a**) lung cancer, (**b**) hematological malignancies, (**c**) breast cancer, (**d**) cancer of the gastro-intestinal tract, and (**e**) renal/urinary tract cancer

Figure 1 illustrates the one-year survival in CAP cases and the age- and sex-matched control group without CAP with the respective cancer subtype. The one-year survival was significantly lower in the control group without cancer across all cancer subtypes, but the survival difference between CAP cases and controls was most pronounced in patients with hematological malignancies, cancer of the gastrointestinal tract, and renal/urinary tract cancer. Of note, the survival difference between CAP cases and controls in lung cancer was small, although the one-year mortality was highest in this group.

The percentage of hospitalized CAP cases with hospital readmissions within 30 days ranged from 27.3% in patients with renal/urinary tract cancer to 44.5% with lung cancer, but readmissions due to CAP were generally less frequent with the highest proportion observed in CAP cases with hematological malignancies (5.8%) (see Additional file 2).

### Predisposing factors of CAP in the nested case-control study

Across all cancer subtypes, men were at higher risk for CAP compared to women and increasing age was a risk factor for CAP. A prior influenza vaccination was associated with a significantly decreased risk of CAP ranging from 56% (breast cancer) to 36% (lung cancer) while pneumococcal vaccination was not associated with a change in risk of CAP in all cancer cohorts. The impact of other and cancer-specific risk factors for CAP varied substantially between the cancer subtypes. Among lung cancer patients, only few risk factors were significantly associated with CAP and their impact on the risk was only small. For patients with hematological malignancies, recent stem cell transplantation substantially increased the risk of CAP with an OR of 7.1 (95%-confidence interval: 4.4–11.5). In women with breast cancer, we observed a nearly 6-fold increased risk for chronic severe liver disease. Among patients with cancer of the gastrointestinal tract and for renal/urinary tract cancer patients, the highest risk was found for patients with metastatic solid tumor with a 3-fold and 4-fold increased risk of CAP, respectively. Predisposing factors of CAP in the multivariate logistic regression models of the nested-case control analysis in the different cancer subtypes are available in Additional file 2.

### Healthcare-related costs in the year after index date for CAP cases and PS-matched controls

Table 3 displays the healthcare-related costs in the year after the index date for CAP cases and PS-matched controls without CAP in the different cancer cohorts. The baseline characteristics of CAP cases and matched controls are available in Additional file 2. The mean overall costs as well as the component-specific mean costs were significantly increased in CAP cases compared to controls across all cancer subtypes. The only exception was the mean ambulatory care costs in patients with hematological malignancies, cancer of the gastrointestinal tract, and renal/urinary tract cancer. The highest costs after the index date were observed in CAP cases with hematological malignancies with 28,969 € (SD 37,142 €) and the lowest in patients with renal/urinary tract cancer with 17,432 € (SD 19,579 €). The absolute difference in the mean overall costs between CAP cases and controls was 4114 € in lung cancer patients, 9826 € in hematological malignancies, 9748 € in breast cancer, 7616 € in cancer of the gastrointestinal tract and 6577 € in renal/urinary tract cancer. These differences were predominantly triggered by substantially elevated hospital costs in CAP cases.

**Table 3:** Healthcare-related costs in the year after index date for CAP cases and PS-matched controls

	CAP	Comparator		
	Mean costs in € (SD)	Mean costs in € (SD)	SMD	*p*-value
Lung cancer	Cases *n* = 1529	Controls *n* = 1529		
Hospital	14,873 (15,154)	11,306 (13,888)	0.25	< 0.001
Ambulatory care	1846 (2651)	1701 (2344)	0.06	0.008
Drug treatment	4037 (7323)	3748 (7611)	0.04	< 0.001
Remedies and aids	592 (1423)	480 (1113)	0.09	0.008
Overall	21,349 (18,366)	17,235 (16,959)	0.23	< 0.001
Hematological malignancy	Cases *n* = 731	Controls *n* = 731		
Hospital	18,417 (32,041)	9452 (22,544)	0.32	< 0.001
Ambulatory care	1762 (2143)	1588 (1636)	0.09	0.669
Drug treatment	8258 (14,315)	7671 (16,793)	0.04	< 0.001
Remedies and aids	532 (1462)	432 (1015)	0.08	0.202
Overall	28,969 (37,142)	19,143 (30,158)	0.29	< 0.001
Breast cancer	Cases *n* = 359	Controls n = 359		
Hospital	8709 (14,101)	2823 (5126)	0.55	< 0.001
Ambulatory care	2438 (2461)	1535 (1634)	0.43	< 0.001
Drug treatment	6074 (11,891)	3310 (8392)	0.27	< 0.001
Remedies and aids	741 (1210)	547 (1035)	0.17	0.02
Overall	17,962 (19,988)	8214 (11,572)	0.60	< 0.001
Cancer of the gastrointestinal tract	Cases *n* = 665	Controls = 665		
Hospital	13,827 (19,832)	7286 (13,957)	0.38	< 0.001
Ambulatory care	1420 (1896)	1287 (1363)	0.08	0.352
Drug treatment	4448 (9923)	3647 (9660)	0.08	< 0.001
Remedies and aids	833 (1834)	691 (1486)	0.09	0.019
Overall	20,527 (23,499)	12,911 (18,539)	0.36	< 0.001
Renal/urinary tract cancer	Cases *n* = 371	Controls 371		
Hospital	12,558 (16,485)	6605 (15,657)	0.37	< 0.001
Ambulatory care	1118 (978)	1283 (1339)	0.14	0.014
Drug treatment	3131 (7697)	2398 (5834)	0.11	0.042
Remedies and aids	624 (1182)	569 (1290)	0.04	0.029
Overall	17,432 (19,579)	10,855 (17,639)	0.35	< 0.001

CAP Community acquired pneumonia, *SD* standard deviation, *SMD* Standardized mean difference

## Discussion

Our study suggests that patients with incident cancer are at a high risk for CAP which is increased compared to patients without cancer in the general population. Lung cancer patients had a 21-fold increased risk compared to the cohort without cancer. For the other cancer subtypes, the risk for CAP was 4.3-fold (hematological malignancies) to 1.7-fold (breast cancer) increased. To our knowledge, this is the first study to examine the epidemiologic and cost burden of CAP in incidence cancer patients by cancer subtype. Pelton et al. [3] investigated the risk of all-cause pneumonia in patients with malignant neoplasm based on German claims data and found a 3.4 to 3.7-fold increased rate ratio in adults with an event rate ranging from 1556 per 100,000 person years in patients aged 18–49 years to 4957 in patients ages 60+ years compared to healthy controls. Unfortunately, they used a different case definition and did not restrict the analysis to patients with incident cancer limiting the comparability to the present study. In another study based on German claims data, Kolditz et al. [11] found that a malignant neoplasms were associated with a 32% increased risk of CAP. In three studies from the United States based on the population-based Surveillance, Epidemiology End Results (SEER)-Medicare database, an elevated risk of pneumonia (27 to 42%) was found for different types of hematological malignancies [12–14].

In addition, our results reveal a high 30-day and one-year mortality after CAP across all cancer subtypes studied in our analysis. The 30-day mortality in CAP cases was highest in lung cancer patients with 20.0% and ranged from 16.9 to 18.5% in CAP cases with other cancer subtypes except for those with breast cancer with only 7.2%. So far, evidence on the mortality after CAP in cancer patients is limited. Kolditz et al. [11] found that CAP cases with malignant neoplasm are at two-fold increased risk of death within 30 days to those without. In our study, the one-year survival was substantially lower for cancer patients with CAP compared to those without, especially in patients with hematological malignancies, cancer of the gastrointestinal tract and renal/urinary tract cancer. The poor survival prognosis of CAP cases may be related to factors associated with CAP, but the occurrence of CAP itself can also be considered as a result of a poor prognosis in cancer patients. Interestingly, the survival difference between CAP cases and controls in lung cancer was very small, although the one-year mortality was highest in this group at 63.5%. This highlights the fact that CAP itself does not have a major impact on survival in the lung cancer group and that the underlying disease itself is the most important predictor of mortality.

Our study showed that CAP is frequently associated with hospital readmissions within 30 days in cancer patients. Exploratory analyses showed that hospital readmissions in general were most often associated with the underlying malignant disease. Possibly, CAP could indirectly lead to complications of the underlying disease and although the CAP was not the direct cause, it may have been part of the causal chain leading to readmission.

In addition, we investigated the impact of predisposing factors for CAP, including pre-defined risk factors according to the German Standing Committee on Vaccination (STIKO) [15] as well as cancer specific risk factors for influenza or pneumococcal infection. As a general finding, we observed a strong variation across different cancer subtypes emphasizing that a differentiation of cancer subtypes (especially lung cancer) is important for studies investigating the burden and risk factors of CAP. An overall pattern unrelated to the cancer subtype was the increased risk of CAP in men compared to women and a higher risk with rising age. This is in line with previous findings from studies investigating risk factors for CAP in the general population [3, 11]. A prior influenza vaccination was associated with a risk reduction for CAP of 56% (breast cancer) to 36% (lung cancer) while pneumococcal vaccination was not significantly associated with CAP in all cancer subtypes. However, this result has to be interpreted with great caution, since our study was not designed to investigate the effectiveness of vaccinations. For instance, severity of disease, i.e. the progression of cancer in our study, was a major confounder in influenza vaccine studies and the probability of death was inversely correlated to the likelihood for the receipt of influenza vaccine [16, 17]. Another important finding was the low impact of pre-defined and cancer-specific risk factors on the CAP risk in lung cancer patients compared to other cancer subtypes. This finding points out that in these patients, the underlying malignant disease itself is the main risk factor for CAP while for other cancer subtypes, co-morbidities such as chronic heart disease or neurological disorders and cancer specific and modifiable risk factors such as immunosuppressant use and cytotoxic chemotherapy are the main predisposing factors. Thus, cancer patients with additional underlying risk factors might especially benefit from preventive measures such as vaccinations. This especially applies for pneumococcal vaccination of patients with hematological malignancies with previous stem cell transplantation who were identified to be at particular risk for CAP in our study. However, there are few data on vaccine efficacy or effectiveness in this patient group so far and the immune response to vaccination in these patients may be impaired [5, 18, 19].

Among patients with a CAP diagnosis, the healthcare-related costs were substantially higher compared to PS-matched controls with cancer and without CAP for each cancer subtype with mean excess costs ranging from 4114 € in lung cancer patients, 9826 € in hematological malignancies, 9748 € in breast cancer, 7616 € in cancer of the gastrointestinal tract and 6577 € in renal/urinary tract cancer. Our study suggests that these excess costs in CAP cases were mainly triggered due to CAP-related hospitalizations. To our knowledge, the costs associated with CAP in cancer patients have not yet been investigated, but previous studies also found excess costs in CAP cases predominantly caused in the hospital setting [20, 21].

### Limitations

Although the analysis dataset obtained from the InGef research database covers more than four million insured members of SHIs all over Germany, representativeness for the whole German population can only be guaranteed with regard to age and sex. Therefore, the CAP incidence in cancer patients and the other outcomes obtained in this study may not be generalizable to the whole German SHI population or patients in other healthcare settings and countries. For instance, selection bias may be introduced if the characteristics of cancer patients in the InGef database systematically differ for instance by socioeconomic status.

As our study does not include a review of individual patient files to confirm the presence or occurrence of cancer as inclusion criterion and CAP as outcome, which for data protection reasons is generally not feasible. Case validation was not possible and misclassification of cancer and CAP cases cannot be ruled out. To minimize the amount of false-positive cases, only primary hospital diagnoses and ambulatory diagnoses in combination with antibiotic prescription and chest imaging were used as case algorithm. Since our study was limited to minimum baseline period of one year, it is possible that we also included some prevalent cancer patients or patients in remission for which no diagnosis was coded in the baseline period; however, we do not expect a substantial impact of this possible misclassification on the incidence rate and other study outcomes. Moreover, preliminary analyses revealed that unspecific ICD-10 GM codes were often used for CAP cases, i.e. the underlying pathogenic agent (e.g. Streptococcus pneumoniae) could not be identified in most cases.

Although we matched CAP cases and controls from the respective cancer cohorts by age, sex, and PS, results for the comparison of costs may be biased by unmeasured confounders not captured in the data due to the observational nature of the study. Despite PS-matching, we observed small differences with slightly higher costs in CAP cases compared to controls. This could reflect that no perfect matching regarding the severity or disease progression of the underlying cancer at baseline was reached. Nevertheless, the excess costs of CAP cases compared to controls in the year after the index date were substantial and cannot be explained by these baseline differences. As a further limitation, it was not possible to differentiate direct costs related to CAP and costs of cancer and other treatments.

## Conclusion

In summary, this study suggests that the incidence rate of CAP and related mortality is high in patients with cancer with strong variations by cancer subtype. Furthermore, CAP in cancer patients is associated with substantial direct excess costs for German SHIs. Given the substantial burden of disease, these results support the need for preventive measures against CAP in this risk group such as vaccinations as one component of a multi-modal prevention strategy. Further research is warranted to investigate the effectiveness of preventive measures as well as the impact of CAP in cancer patients from a clinical and patient perspective.

## Additional files

Additional file 1: Operational definition of inclusion and exclusion criteria, at-risk, high-risk conditions and other risk factors for CAP, vaccinations and cancer specific risk factors for CAP (DOCX 17 kb)

Additional file 2: Summary of additional results (XLSX 128 kb)

## Abbreviations

ATC: Anatomical Therapeutic Classification
CAP: Community-acquired pneumonia
CT: Computed tomography
EBM: German uniform assessment standard
HIV: Human immuno-deficiency virus
HRI: Health Risk Institute
ICD-10 GM: International Classification of Diseases 10th Revision German Modification
InGef: Institute for Applied Health Research Berlin
Morbi-RSA: German structure compensation scheme
MRI: Magnetic Resonance Imaging
OPS: German Procedure Classification
OR: Odds ratio
PS: Propensity score
SD: Standard deviation
SHI: Statutory health insurance providers
sIR: Age- and sex-standardized incidence rates
SMD: Standardized differences
STIKO: German Standing Committee on Vaccination
