# Supplementary material for: Burden of community-acquired pneumonia, predisposing factors and health-care related costs in patients with cancer
Source: BMC Health Serv Res. 2019 Jan 14;19:30. doi: 10.1186/s12913-018-3861-8 (PMC6332528; doi:10.1186/s12913-018-3861-8)
Supplement: Supplementary file 1 — Operational definition of inclusion and exclusion criteria, at-risk, high-risk conditions and other risk factors for CAP, vaccinations and cancer specific risk factors for CAP (DOCX 17 kb) [file 12913_2018_3861_MOESM1_ESM.docx]

**Table 1 Cancer diagnoses used as inclusion criteria**

| **Variable** | **Operational definition** |
| --- | --- |
| Patients with incident diagnosis of the following cancer subtypes:  lung cancer & cancer of airways, hematological malignancies, breast cancer, cancer of the gastro-intestinal tract and renal cancer & cancer of the urinary tract | Patients with a primary or secondary hospital diagnosis **OR** a verified ambulatory diagnosis of the following cancer subtypes:   1. Lung cancer & cancer of airways (ICD-10 GM codes C33x, C34x, C39x) 2. Hematological malignancies (ICD-10 GM codes C81x-C86x, C88x, C90x-C95x) 3. Breast cancer (ICD-10 GM codes C50x) 4. Cancer of the gastro-intestinal tract (ICD-10 GM codes C15x-C21x, C26x) 5. Renal cancer & cancer of the urinary tract (ICD-10 GM codes C64x-C68x) |

**Table 2 Definition of at-risk, high-risk conditions and other risk factors for CAP**

| **Variable** | **Operational definition** |
| --- | --- |
| **At-risk coditions** | |
| Chronic heart disease | Patients with a primary or secondary hospital diagnosis or verified ambulatory diagnosis I05x-I09x, I11x, I13x, I20x, I21x, I22x, I24x, I251, I252, I27x, I34x-I39x, I42x, I50x, I23x, Q20x-Q24x, Q251 |
| Chronic pulmonary disease (incl. asthma) | Patients with a primary or secondary hospital diagnosis or verified ambulatory diagnosis E84x, I278, I279, J40x-J47x, J60x-J65x, J684, J84x, J96x, P27x |
| Diabetes | Patients with a primary or secondary hospital diagnosis or verified ambulatory diagnosis E10x-E14x, P701, T383, G590, G632 |
| Neurological disorders | Patients with a primary or secondary hospital diagnosis or verified ambulatory diagnosis G041, G114, G240, G242, G40x, G41x, G71x, G72x, G80x, P90x, R56x, G81x, G82x, G830-G834, G839 |
| **High-risk conditions** | |
| Autoimmune disease | Patients with a primary or secondary hospital diagnosis or verified ambulatory diagnosis M05x, M06x, M32x, K50x |
| Functional or anatomic asplenia, sickle cell diseases and other hemoglobinopathies | Patients with a primary or secondary hospital diagnosis or verified ambulatory diagnosis Q890, Q893, D56x, D57x, D582, D60x, D61x, D730, D731, D738 |
| HIV-infection | Patients with a primary or secondary hospital diagnosis or verified ambulatory diagnosis B20x-B23x, B24x, Z21x |
| Chronic renal failure or dialysis | Patients with a primary or secondary hospital diagnosis or verified ambulatory diagnosis I120, I131, I132, N01x, N03x, N04x, N05x, N18x, N19x, Q60x, Z49x, Z940, Z992 OR an OPS code (8853x, 8854x, 8855x, 8857x) or EBM code (13602, 13610, 13611, 40823-40828) in the year prior to cohort entry |
| Chronic severe liver disease | Patients with a primary or secondary hospital diagnosis or verified ambulatory diagnosis K70x (excl. K700), K713-K718, K721-K729, K73x, K74x, K750, K751, K762-7, I850, I859 |
| Solid organ or stem cell transplantation | Patients with a primary or secondary hospital diagnosis or verified ambulatory diagnosis Z940-Z944, Z9480, Z9481 or with an OPS code 5411x, 8805x, 5335x, 5375x, 5504x, 5555x |
| Congenital immunodeficiency | Patients with a primary or secondary hospital diagnosis or verified ambulatory diagnosis D80x-D84x, D89x, D71x |
| Neutropenia/agranulocytosis | Patients with a primary or secondary hospital diagnosis or verified ambulatory diagnosis D70x |
| Cochlear implant | Patients with a primary or secondary hospital diagnosis or verified ambulatory diagnosis Z962 or an OPS-code 52092x, 52097x, 52098x |
| Immunosuppressive treatment | Patients with at least one prescription of a drug with the ATC code L04 |
| **Other risk factors** | |
| Hospitalizations due to pneumonia | Patients with a hospitalization with a primary hospital diagnosis of pneumonia (ICD-10 codes J100, J110, J12x-J18x) |
| Residence in residential care or nursing home | Patients with EBM codes 01415, 14314, 16231, 21231 OR a hospital discharge cause 10 „Entlassung in eine Pflegeeinrichtung“ |

**Table 3 Definition of Vaccinations**

| **Variable** | **Operational definition** |
| --- | --- |
| Pneumococcal vaccination | Patients with EBM codes 89118x, 89119, 89120 (assessed within 5 years before the index date in the nested case-control analysis |
| Influenza vaccination | Patients with EBM codes 89111 or 89112x (assessed within 6 months before the index date in the nested case-control analysis |

**Table 4 Definition of cancer specific risk factors for CAP**

| **Variable** | **Operational definition** |
| --- | --- |
| Metastatic solid tumor | Patients with a primary or secondary hospital diagnosis or verified ambulatory diagnosis C77x-C79x (yes vs. no)  (not considered for haematological cancer) |
| Radiation therapy | Patients with an inpatient OPS-code indicating radiation therapy (OPS code 852x) **OR** a primary or secondary hospital diagnosis indicating radiation therapy (ICD-10 GM code Z510, Z5182) **OR** an EBM-code 25320-25323, 25330-25333)  Patients were classified into the following mutually exclusive groups based on their last claim for radiation therapy:   - Radiation therapy 0-<90 days prior to the index date - No claim for radiation therapy |
| Cytotoxic chemotherapy | Patients with an inpatient OPS code indicating cytotoxic chemotherapy (OPS code 8542x, 8543x, 85440, 85441) **OR** a primary or secondary hospital diagnosis indicating chemotherapy therapy (ICD 10 GM code Z511, Z5182) **OR** at least one prescription of drug with the three digit ATC code L01 (antineoplastic agents).  Patients were classified into the following mutually exclusive groups based on their last claim for cytotoxic chemotherapy:   - Cytotoxic chemotherapy 0-<90 days prior to the index date - No claim for cytotoxic chemotherapy |
| Use of immunosuppressants incl. corticosteroids for systemic use | Patients with at least one prescription of a drug with the ATC code L04 (immunosuppressants) as well as H02A, H02B (corticosteroids for systemic use) **OR** with an ambulatory or inpatient OPS code indicating immunosuppression (OPS Codes 85470, 85473)  Patients were classified into the following mutually exclusive groups based on their last prescription:   - Immunosuppressant use 0-<90 days prior to the index date - No immunosuppressant use |
| Stem cell transplantation (for haematological malignancies only) | Patients with a primary or secondary hospital diagnosis or verified ambulatory diagnosis Z9480, Z9481 or with an OPS code 5411x, OR 8805x Patients were classified into the following mutually exclusive groups based on their last prescription:   - Stem cell transplantation 0-<90 days prior to the index date - No Stem cell transplantation |
